# Diagnostic Developments in Differentiating Unresponsive Wakefulness Syndrome and the Minimally Conscious State

**DOI:** 10.3389/fneur.2021.778951

**Published:** 2022-01-13

**Authors:** Camillo Porcaro, Idan Efim Nemirovsky, Francesco Riganello, Zahra Mansour, Antonio Cerasa, Paolo Tonin, Bobby Stojanoski, Andrea Soddu

**Affiliations:** ^1^Department of Neuroscience and Padova Neuroscience Center (PNC), University of Padova, Padova, Italy; ^2^Institute of Cognitive Sciences and Technologies (ISTC)–National Research Council (CNR), Rome, Italy; ^3^Department of Information Engineering, Università Politecnica delle Marche, Ancona, Italy; ^4^Centre for Human Brain Health, School of Psychology, University of Birmingham, Birmingham, United Kingdom; ^5^Department of Physics and Astronomy, Brain and Mind Institute, University of Western Ontario, London, ON, Canada; ^6^Sant'Anna Institute and Research in Advanced Neurorehabilitation (RAN), Crotone, Italy; ^7^Institute for Biomedical Research and Innovation (IRIB), National Research Council, Messina, Italy; ^8^Pharmacotechnology Documentation and Transfer Unit, Preclinical and Translational Pharmacology, Department of Pharmacy, Health Science and Nutrition, University of Calabria, Rende, Italy; ^9^Faculty of Social Science and Humanities, University of Ontario Institute of Technology, Oshawa, ON, Canada; ^10^Department of Psychology, Brain and Mind Institute, University of Western Ontario, London, ON, Canada

**Keywords:** vegetative state (VS), minimally conscious state (MCS), unresponsiveness wakefulness syndrome (UWS), disorder of consciousness (DOC), magneto-electroencephalography (M-EEG), functional magnetic resonance imaging (fMRI), positron emission tomography (PET), transcranial magnetic stimulation (TMS)

## Abstract

When treating patients with a disorder of consciousness (DOC), it is essential to obtain an accurate diagnosis as soon as possible to generate individualized treatment programs. However, accurately diagnosing patients with DOCs is challenging and prone to errors when differentiating patients in a Vegetative State/Unresponsive Wakefulness Syndrome (VS/UWS) from those in a Minimally Conscious State (MCS). Upwards of ~40% of patients with a DOC can be misdiagnosed when specifically designed behavioral scales are not employed or improperly administered. To improve diagnostic accuracy for these patients, several important neuroimaging and electrophysiological technologies have been proposed. These include Positron Emission Tomography (PET), functional Magnetic Resonance Imaging (fMRI), Electroencephalography (EEG), and Transcranial Magnetic Stimulation (TMS). Here, we review the different ways in which these techniques can improve diagnostic differentiation between VS/UWS and MCS patients. We do so by referring to studies that were conducted within the last 10 years, which were extracted from the PubMed database. In total, 55 studies met our criteria (clinical diagnoses of VS/UWS from MCS as made by PET, fMRI, EEG and TMS- EEG tools) and were included in this review. By summarizing the promising results achieved in understanding and diagnosing these conditions, we aim to emphasize the need for more such tools to be incorporated in standard clinical practice, as well as the importance of data sharing to incentivize the community to meet these goals.

## Introduction

Consciousness remains one of the most challenging phenomena to understand, and, not surprisingly, an objective and generally agreed-upon definition of consciousness remains elusive (1). In common terms, consciousness is described based on observations of wakefulness (i.e., the presence of spontaneous periods of eye-opening) and awareness (i.e., the ability for a subject to respond to internal/external stimuli coherently). However, clinical research employing advanced neuroimaging tools continues to underscore the intricate aspects of this concept, and it is now well-established that consciousness cannot be described with external observations of behavior alone.

**Figure 1:**
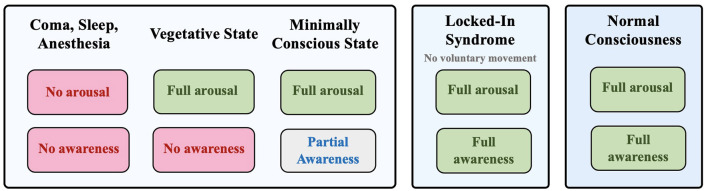
Different states of consciousness: coma, vegetative state, minimally conscious state, locked-in syndrome, and normal consciousness based on the degree of arousal and awareness.

One essential area of research on consciousness involves a group of pathologies known as disorders of consciousness (DOC). Diagnosis of DOC pertains to patients who lose the ability to demonstrate overt behaviors due to traumatic brain injury (1), and there are four major DOC categories they may be ascribed to: 1) Coma, 2) Vegetative State (VS), also known as Unresponsive Wakefulness Syndrome (UWS) (2), 3) Minimally Conscious State (MCS), and 4) Locked-in Syndrome (LIS) (3) (Figure 1). In recent years, neuroimaging studies on DOC individuals challenged previous assumptions about their conditions. Most notably, it was found that they can maintain varying levels of residual cognitive capabilities that are not reflected through demonstrated behavior (4). Accordingly, further terminology has been adopted to describe conditions related to DOC, including cognitive motor dissociation (CMD) and higher-order cortex motor dissociation (HMD), which stress the presence of cortical function despite the absence of behavioral capacity (5, 6).

In accordance with findings that underscored the sophisticated pathology of these disorders, properly diagnosing patients with DOC is a challenging process. Nevertheless, an accurate diagnosis is crucial to providing effective treatments and improving the likelihood of recovery for these patients.

With these important motivations, this review is primarily focused on the two most misdiagnosed DOC: the Vegetative State/Unresponsive Wakefulness Syndrome (VS/UWS) and the Minimally Conscious State (MCS). Although VS/UWS patients demonstrate behavioral sleep/wake cycles and may maintain cortical functions associated with awareness, they are otherwise unresponsive to their external environment (7). On the other hand, individuals in MCS show some level of overt awareness and an ability to follow commands, albeit inconsistently (8).

So far, several reviews provided comprehensive summaries about findings on DOC neurophysiology, diagnosis, and treatment (9, 10). More recently, focus was also given to prognostic factors related to the recovery of consciousness, especially in patients who transition from VS/UWS to MCS (11). Although DOC misdiagnosis is widely discussed in clinical neuroscience, a comprehensive consolidation of the tools that can be used to solve this problem is lacking. Therefore, the purpose of the present review is to thoroughly integrate and highlight the important ways in which neuroimaging and electrophysiological techniques can be used to accurately differentiate VS/UWS and MCS patients.

To collect information on how neuroimaging and electrophysiological techniques have been employed to distinguish the two conditions, we queried the PubMed database for relevant studies conducted in the last 10 years. This led to 144 publications (39 reviews and 105 research articles), which we narrowed down to 55 papers that focused on differentiating VS/UWS from MCS using tools such as PET, fMRI, EEG, and TMS-EEG.

Functional neuroimaging techniques, such as Positron Emission Tomography (PET) (7, 12) and functional Magnetic Resonance Imaging (fMRI) (13–15), have been introduced as means to assess patients with a DOC. Going beyond assessments that rely on behavioral observations, such as the Coma Recovery Scale-Revised (CRS-R), these techniques allow for an analysis of brain activity in response to certain mental tasks that do not require overt behavior, which have proven to be useful tools to diagnose patients as MCS or VS/UWS (4, 16). Despite the many advantages of using PET and fMRI, these techniques also have limitations as clinical tools; in particular, both methods are very costly and not easily available (17).

Electroencephalography (EEG), a technique used to measure the activity of cortical neurons using electrodes on the scalp, has emerged as a viable alternative to PET and MRI. First, EEG has the advantage of being less costly and easier to administer. It is also more adaptable for longitudinal studies and there are very few exclusion criteria for patients. Perhaps most importantly, these advantages make EEG employable at the bedside (17, 18). This eliminates the requirement for patients to be transferred to a suitably equipped fMRI/PET facility, making EEG especially useful in studies on DOC (19). Moreover, EEG data can be monitored and analyzed in real-time at the bedside to compute complex parameters that provide a quantitative evaluation of brain signals, a technique called quantitative EEG (qEEG) (20). qEEG consists of numerical computations of EEG parameters such as power spectra (21) or entropy (22), which have been used to reliably differentiate VS/UWS from MCS patients.

While introducing neuroimaging (23) and electrophysiological techniques (24, 25) to clinical settings has reduced misdiagnosis in cases involving DOC patients, the reliability of these methods can vary significantly across studies/clinics and their implementation requires further research before they can constitute standard clinical practice (23–25).

Each section of this review will focus on how a specific neuroimaging or electrophysiological technique can help distinguish VS/UWS and MCS patients. Moreover, we discuss several advantages, disadvantages, and other important factors to consider for each method. Beyond all the important progress already made, we aim to motivate further research into the development of these methodologies so that more tools can be adopted in standard clinical practice.

**Figure 2:**
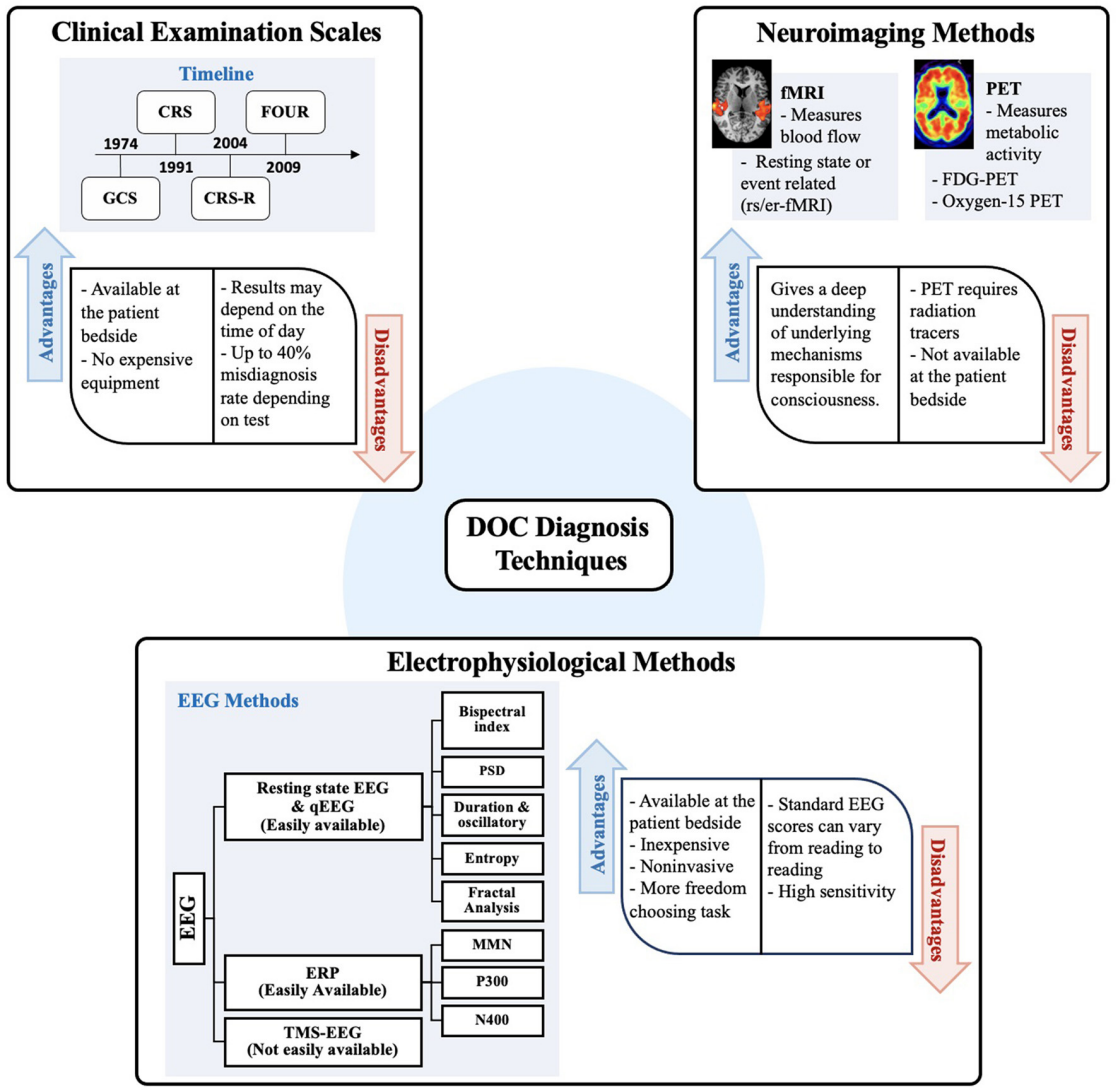
This schematization summarizes the main methods used to distinguish diagnosis between VS/UWS and MCS. Clinical examination scales are illustrated using a timeline indicating implementation or modification of existing scales since 1974. Next, neuroimaging methods commonly used such as PET and fMRI in DOC patients are listed. Finally, electrophysiological methods and different approaches for feature extraction from EEG are listed as well as different tasks implemented by using EEG. Advantages and disadvantages are also summarized for each method. GCS, Glasgow coma scale; CRS, Coma recovery scale; CRS-R, Coma recovery scale-revised; FOUR, Full outline of unresponsiveness; STAR, Sensory tool to assess responsiveness; EEG, electroencephalogram; QEEG, Quantitative analysis of electroencephalogram; ERP, Event-Related potential; MMN, Mismatch negativity; TMS, Transcranial magnetic stimulation.

For a summary of the primary techniques reviewed here, see Figure 2. The main clinical scales are summarized in Table 1. While, methods and results of research articles that used neuroimaging techniques are summarized in Table 2 (PET and fMRI) and those that explored electrophysiological tools are listed in Table 3.

**Table 1:** Assessment scales overview.

**Scale**	**Behavioral content**	**Standardized administration and scoring**	**Content validity aspen criteria**	**Diagnostic validity**	**Prognostic validity**	**Public domain**	**Estimate time require (minutes)**
CRS-S (26)	Auditory, visual, motor, oral, communication, arousal	Acceptable	Excellent	Unproven (class IV)	Unproven (not studied)	YES	25
SMART (27)	Auditory, vision, tactile, olfactory, gustatory, wakefulness, motor, communication	Acceptable	Good	Unproven (not studied)	Unproven (class IV)	NO	60+
WNSSP (28)	Visual, tactile, olfactory, arousal/attention, auditory, expressive communication	Acceptable	Good	Unproven (not studied)	Unproven (class IV)	YES	45
SSAM (29)	Auditory, vision, tactile, olfactory, gustatory, eye-opening, motor, vocalization	Acceptable	Good	Unproven (not studied)	Unproven (not studied)	YES	30
WHIM (30)	Basic behaviors, social/communication, attention/cognitive, orientation/memory	Acceptable	Good	Unproven (not studied)	Unproven (not studied)	NO	30–120
DOCS (31)	Auditory, visual, tactile, sensory, swallowing, olfactory	Acceptable	Acceptable	Unproven (not studied)	Unproven (class IV)	YES	45
FOUR (32)	Eye response, motor response, respiration, brainstem reflexes	Not acceptable	Unacceptable	Unproven (class IV)	Probably predictive (class I)	YES	10
CNC (33)	Visual, auditory, command following, threat response, olfactory, tactile, pain, vocalization	Acceptable	Acceptable	Unproven (not studied)	Unproven (class IV)	YES	10
STAR (34)	Visual, auditory, motor, communication, emotion	Acceptable	Good	Unproven (not studied)	Unproven (not studied)	NO	6–23

*CRS-R, Coma recovery scale-revised; SMART, Sensory modal assessment rehabilitation technique; WNSSP, Western neuro stimulation profile; SSAM, Sensory stimulation assessment measure; WHIM, Wessex head injury; DOCS, Disorders of consciousness scale matrix*.

***Levels of evidence for diagnostic studies: Class I**, cohort survey with prospective data collection, includes a broad spectrum of people at risk of developing the outcome of interest, employs objective outcome assessment, inclusion criteria are well-defined, and at least 80% of people enrolled have the risk factor and the outcome measure; **Class II**, cohort study with retrospective data collection or case-control study, includes a broad spectrum of people with and without the risk factor and the outcome, and the risk factor and outcome are determined objectively and independently; **Class III**, cohort or case-control studies that include a narrow spectrum of patients with or without the disease in which the diagnostic test result and disease status are determined objectively and independently or by different investigations; **Class IV**, studies that do not include people suspected or known to have or not have the disease, the reference standard is undefined or not independent, and there are no calculable measures of diagnostic accuracy or precision. For further information about evidence classification, the interested reader is referred to the American Academy of Neurology Guideline Process Manual (35)*.

**Table 2:** Studies reviewed and included in this review.

**Neuroimaging studies**						
**No**	**References**	**Sample**	**Method**	**Clinical rating**	**Task**	**Results**
**Positron emission tomography (PET)**						
1	Stender et al. (36)	102 DOC patients (26 VS/UWS, 76 MCS)	FDG-PET	CRS-R	Motor and visuospatial imagery	VS/UWS showed complete bilateral hypometabolism of the associative frontoparietal cortex and no voxels with preserved metabolism. MCS showed incomplete hypometabolism and partial preservation of activity within the frontoparietal cortex
2	Stender et al. (36)	41 DOC patients (14 VS/UWS, 21 MCS, 6EMCS) 29 healthy participants	FDG-PET	CRS-R	Resting-State	Cerebral metabolic rate of glucose (CMRglc) reduction was observed for both VS/UWS (42%) and MCS (55%) with respect to healthy controls. It was proposed that the majority of the differences between VS/UWS and MCS are concentrated in the fronto-parietal cortex and thalamus
3	Laureys et al. (7)	5 DOC patients (5 VS/UWS, 0MCS) 18 healthy participants	FDG-PET	ANA	Auditory	VS/UWS patients failed to activate the temporoparietal junction cortex but activation in the auditory cortex was preserved bilaterally
4	Toyoshima et al. (37)	6 DOC patients (emerged from VS/UWS to MCS) 30 healthy participants	FDG-PET	CSR-R	Speech	In the patient group, a significant reduction in glucose metabolism was observed in the cerebral peduncles and bilaterally in the cingulum
5	Hermann et al. (38)	52 DOC patients (21 VS/UWS, 31 MCS)	FDG-PET & qEEG	CRS-R	Oddball paradigm	Adoption of qEEG alongside FDG-PET significantly improved diagnostic accuracy compared to using PET or qEEG individually (81% and 88% +/– predictive values for MCS)
**Event-Related fMRI**						
6	Vogel et al. (39)	22 DOC patients (10 VS/UWS, 12 MCS)	fMRI	CRS-R	Mental imagery	Higher BOLD activation was observed in the mental imagery regions of 4 VS/UWS and 9 MCS patients. Improvement of consciousness was observed for all the patients who responded to tasks
7	Crone et al. (40)	25 DOC patients (17 VS/UWS, 8MCS) 25 healthy participants	fMRI	CRS-R	Auditory	No activation of DMN was observed in the medial regions of the VS/UWS patients, while there was a reduction in MCS patients compared to healthy subjects
8	Okumura et al. (41)	7 DOC patients (5 VS/UWS, 2 MCS) 21 healthy participants	fMRI	CRS	Music stimulation	In all healthy and MCS patients, activation was observed in the bilateral superior temporal gyri, but not in the VS/UWS patients
9	Monti et al. (14)	24 DOC patients (8 VS/UWS, 16 MCS, 4 EMCS) 16 healthy participants	fMRI	CRS-R	Auditory and visual	Different BOLD activations were observed for all the patients who have clear connectivity between the prefrontal cortex and the anterior thalamus (3 VS/UWS and 6 MCS)
10	Stender et al. (13)	122 DOC patients (41 VS/UWS, 81 MCS)	fMRI	CRS-R	Motor and visuospatial imagery	It was shown that the use of event-related fMRI to compare activity patterns during motor imagery tasks and rest couldn't be considered as a sensitive tool to distinguish between VS/UWS and MCS patients
11	Marino et al. (42)	50 DOC patients (23 VS/UWS, 27 MCS)	fMRI	GCS	Auditory	Significant BOLD activation in the primary auditory cortex was observed in both MCS and VS/UWS with higher activation in MCS. Among the VS/UWS, 10 patients with responses similar to the MCS group transitioned into MCS later on
**Resting-State fMRI**						
12	Demertzi et al. (43)	48 DOC patients (24 VS/UWS, 24 MCS) 57 healthy participants	fMRI	CRS-R	Resting-State	Default mode network (DMN) was reduced in patients with respect to healthy subjects with 85% of accuracy. However, no obvious difference emerged between VS/UWS and MCS patients
13	Vanhaudenhuyse et al. (44)	14 DOC patients (4 VS/UWS, 4 MCS, 5 coma, 1 LIS) 14 healthy participants	fMRI	CRS-R	Resting-state	Decreased DMN connectivity in DOC correlated with the degree of consciousness impairment. Moreover, impairment in the precuneus connectivity was more significant in VS patients than MCS
14	Di Perri et al. (45)	18 DOC patients (11 VS/UWS, 7 MCS) 18 healthy participants	fMRI	CRS-R	Resting-state	DMN hypoconnectivity associated with hyperconnectivity limbic structures was found in DOC patients. Hyperconnectivity may reinforce neural loops and disrupt the normal pattern of connectivity
15	Lutkenhoff et al. (46)	115 DOC patients (38 VS/UWS, 63 MCS, 14 EMCS) 96 healthy participants	fMRI	CRS-R	Resting-state	In DOC patients, awareness and wakefulness were associated with tissue atrophy within thalamic and basal ganglia nuclei, respectively, which were inversely correlated with the consciousness level. No significant differences found among patient groups
16	Long et al. (47)	18 DOC patients (11 VS/UWS, 7 MCS) 13 healthy participants	fMRI	CRS-R	Resting-state	In DOC patients, the combination of topological patterns observed in the FPN and DMN showed a better ability to predict consciousness compared to other networks
17	Di Perri et al. (48)	58 DOC patients (21 VS/UWS, 24 MCS, 13 EMCS) 35 healthy participants	fMRI	CRS-R	Resting-state	Positive DMN differentiated heathy from DOC patients but not VS/UWS from MCS. Negative DMN was observed only in healthy subjects and EMCS
18	Fernández-Espejo et al. (49)	52 DOC patients (19 VS/UWS, 27 MCS,6 EMCS) 23 healthy participants	fMRI	CRS-R	Resting-state	Congruence between posterior areas of the DMN correlated with the level of consciousness, which was higher in MCS than VS/UWS patients
19	Kondziella et al. (50)	7 DOC patients (1 VS/UWS, 3 MCS, 1 EMCS,2C)	fMRI	CRS-R	Resting-state	Preserved DMN activity correlated with the recovery of DOC patients. The structural integrity of this network correlated with behavioral signs of consciousness. Normal DMN was found in one MCS patient
20	Demertzi et al. (51)	101 DOC patients (32 VS/UWS, 59 MCS) 58 healthy participants	fMRI	CRS-R	Resting-state	Measures of inter-regional activity coordination were used to identify patterns associated with VS/UWS (low coordination pattern) and wakefulness (long-range coherence pattern)
21	Varley et al. (52)	18 DOC patients (8VS/UWS, 10 MCS) 15 healthy participants	fMRI	Not indicated	Resting-state	Fractal values were associated with the level of consciousness, with higher fractal (i.e., more complex) networks being associated with higher levels of consciousness

*The table shows the main results reported in the literature reviewed for the neuroimaging methods*.

**Table 3:** Studies were reviewed and included in the review.

**Electrophysiological methods**						
**No**	**References**	**Sample**	**Method**	**Clinical rating**	**Task**	**Result**
**Resting-state/Sleep and qEEG**						
1	Lehembre et al. (21)	31 DOC patients (10 VS/UWS, 21MCS)	EEG (power spectrum)	CRS-R	Resting state	Increases in delta and alpha power were observed in VS/UWS subjects with respect to MCS. Moreover, a connectivity reduction particularly, in alpha and theta bands, was also observed
2	Bagnato et al. (53)	46 DOC patients (25 VS/UWS, 21 MCS)	EEG (SYNEK scale) 21 electrodes	LCF	Resting-state	A correlation between the SYNEK score and the LCF scale was observed in patients with non-traumatic brain injuries but not in those with traumatic brain injuries
3	Fingelkurts et al. (54)	21 DOC patients (14 VS/UWS, 7 MCS)	EEG (duration & oscillatory) 21 electrodes	ANA	Resting-state	A relation between the level of consciousness and the dynamic parameters of EEG microstates such as the duration and oscillation were observed Oscillation was reduced by 50% compared to normal subjects The reduction in the number of alpha rhythm spectral patterns was 37% for MCS and 26% for VS/UWS
4	Lechinger et al. (55)	17 DOC patients (8 VS/UWS, 9 MCS)	EEG (power spectrum) 19 electrodes	CRS-R	Resting-state	VS/UWS patients showed higher values in both theta and delta average relative resting amplitude, while no difference has been shown between controls and MCS patients
5	Bonfiglio et al. (56)	9 DOC patients (4 VS/UWS, 5 MCS) 12 healthy participants	EEG 19 electrodes	CRS-R	Resting-state	Reduction in the intensity of the Blink-Related Synchronization/Desynchronization (nBRS/BRD), particularly on the inferior and temporo-occipital junction, was observed in VS/UWS patients with respect to the control group. MCS patients showed intermediate intensity
6	Schnakers et al. (57)	43 DOC patients (13 VS/UWS, 30 MCS)	EEG (BIS)	GCS, CRS-R	Resting-state	The Bispectral index (BIS) showed the highest correlation with the level of consciousness. Moreover, the BIS was able to predict the probability the patients recovering
7	Engemann et al. (58)	327 DOC patients (148 VS/UWS, 179 MCS) 66 healthy participants	EEG (power spectrum & more)	CRS-R	Resting-state and “Local-Global” Protocol	A broad machine-learning classification analysis showed that several EEG-derived features can accurately discriminate MCS and VS/UWS patients in a robust manner (different acquisition methodologies, clinical settings)
8	Gosseries et al. (59)	56 DOC patients (24 VS/UWS, 26 MCS, 6 coma)	EEG (Entropy) 3 electrodes	CRS-R	Resting-state	VS/UWS patients showed higher response entropy (73 ± 19) with respect to MCS patients (45 ± 28)
9	Piarulli et al. (60)	12 DOC patients (6 VS/UWS, 6 MCS)	EEG (spectral entropy) 12 electrodes	CRS-R	Resting-state	Lower delta, higher theta and alpha power, and higher spectral entropy was observed in MCS (0.68) with respect to VS/UWS patients (0.59). Moreover, periodicity was absent in VS/UWS while MCS patients showed periodicity similar to healthy subjects
10	Marinazzo et al. (61)	26 DOC patients (11 VS/UWS, 5 MCS, 5 EMCS) 10 healthy participants	EEG (transfer entropy) 19 electrodes	CRS-R	Resting-state	Transfer entropy was able to indicate EMCS easily among DOC patients, but the procedure was not as useful for other patient classifications It was also found that information transfer increased for DOC patients in centrals region but decreased in the lateral regions
**Event-Related potential ERP**						
11	Cavinato et al. (62)	17 DOC patients (6 VS/UWS, 11 MCS) 10 healthy people	ERP (P300)	CRS-R	Auditory	Cortical response was detected in the MCS patients due to the stimulation. The increase of P300 latency was observed only in MCS patients and healthy controls
12	King et al. (22)	181 DOC patients (75 VS/UWS, 68 MCS, 24 Coma) 14 healthy participants	EEG WSMI	CRS-R	Auditory	A novel metric called Weighted Symbolic Mutual Information (WSMI) was employed. WSMI across distant cortical and thalamic areas showed a correlation with the level of consciousness. For VS patients, WSMI in the frontal area was less impaired than in the posterior regions
13	Wang et al. (63)	11 DOC patients (6 VS/UWS, 5 MCS) 5 healthy participants	ERP (P300, MMN)	CRS-R	Auditory	MMN and P300 were observed in all MCS patients and in 5/6 VS/UWS patients. P300 was observed in 4/6 VS/UWS patients
14	Calabrò et al. (64)	21 DOC patients (11VS, 10MCS)	ERP (LORETA) 19 electrodes	CRS-R	Heat stimuli	Partially preserved gamma band ERP activation was observed in MCS patients, and in only 2/11 VS/UWS patients
15	Leo et al. (65)	22 DOC patients (10VS, 12MCS)	ERP 6 electrodes	CRS-R	Heat stimuli	Changes in the HRV and oxygen saturation in response to stimulation were observed in MCS patients, while no change was observed in VS/UWS patients (with the exception of two such individuals)
16	Boly et al. (66)	21 DOC patients (8 VS, 13MCS)	ERP MMN 60 electrodes	CRS-R	Auditory	Significant impairments of backward connectivity (from frontal to temporal) during a mismatch negativity paradigm was observed in VS/UWS patients
17	Sitt et al. (67)	167 DOC patients (75 VS/UWS, 68 MCS, 24 coma) 24 healthy people	ERP (P300, MMN) 56-electrode geodesic sensor net (EGI)	CRS-R	Auditory	P300 and MMN were found to differentiate between patients and healthy controls but not between DOC groups with acceptable accuracy, while EEG complexity could be the most acceptable indicator
18	Raimondo et al. (68)	127 DOC patients (70 VS/UWS, 50 MCS)	ERP Correlate with HR	CRS-R	Auditory	Only MCS patients showed a phase shift in a cardiac cycle after auditory stimulation, represented by a significant short interval between the auditory stimulation and the following R peak
19	De Biase et al. (69)	62 DOC patients (57 VS/UWS, 5 MCS)	ERP (REM) 19 electrodes	CRS-R and GCS	Polysomnography (PSG) Somatosensory Auditory Visual	Patients who showed all sleep elements had higher CRS-R value (7/62) Polysomnography recordings have confirmed to be a reliable tool in the neurophysiologic assessment of patients suffering from prolonged DOCs (more adequately than EPs)
20	Faugeras et al. (70)	49 DOC patients (22 VS/UWS, 19 MCS, 8coma) 10 healthy people	ERP (MMN, global and local effect) 256 electrodes	CRS-R	Auditory	13/19 MCS patients showed a response to P3a and P3b (global effect) while only 2/22 VS/UWS patients showed the same global effect
21	Kotchoubey et al. (71)	98 DOC patients (60 PVS, 38 MCS)	ERP (MMN, P300, N1) 9 electrodes	CRS-R	Auditory	MCS and VS/UWS showed a significant difference only in MMN frequency (*p* < 0.05) while no difference was observed for N1 and P300
22	Rohaut et al. (72)	30 DOC patients (15 VS/UWS, 15 MCS) 20 healthy participants	ERP (LPC, N400) 256 electrodes	CRS-R	Auditory	The LPC (late positive components) response was only observed in MCS and healthy groups. The LPC was proposed to be an indicator of a potential specific marker of conscious semantic processing. The only DOC patients (3/30) who showed significant LPC and N400 components were MCS
23	Rivera-Lillo et al. (73)	13 DOC patients (VS/UWS & MCS) 10 healthy participants	ERP (P300) 24 electrodes	CRS-R	Auditory	A correlation between the strength of the P300 and the percentage of epochs with delta event-related synchronization (ERS) was found
24	Balconi et al. (74)	18 patients (10 VS/UWS, 8 MCS) 20 healthy participants	ERP (N400) 64 electrodes	GSC	Auditory	Increasing peak amplitude of N400 within the fronto-central cortical areas was found in reaction to incongruous sequences for both controls and DOC patients. Thus, semantic processing was partially preserved in both MCS and VS/UWS patients
25	Braiman et al. (75)	21 DOC patients (3 VS/UWS, 12 MCS, 6 EMCS) healthy people	ERP natural speech envelope correlates (NSE)	CRS-R	Auditory	The earliest NSE reactions was observed in healthy controls, while delayed latency reaction was observed in the VS/UWS and MCS patient groups
26	Estraneo et al. (76)	143 DOC patients (70 VS, 73MCS)	P300 19 electrodes	CRS-R	Eye opening and closing, tactile, noxious, acoustic, and Intermittent photic stimuli	The patients who showed response to auditory stimuli and forced eye closing tasks had a higher probability of showing improvement of their condition. The other tasks failed to predict future improvement
27	Annen et al. (77)	40 DOC patients (15 VS, 23 MCS, 2 EMCS) 12 healthy participants	P300 EEG based BCI system	CRS-R	Auditory and somato-sensory stimulation	P300 did not show a significant difference between VS and MCS patients. Also, multimodal recordings showed better performance than unimodal assessments in BCI application
28	Binder et al. (78)	15 DOC patients (No information on severity level)	Low and high gamma frequencies (64 electrodes)	CRS-R	Auditory	A strong correlation was found between low gamma range frequencies and CRS-R score. There was also evidence of differences in phase locking indices between VS and MCS in the frequency range between 36 and 47 Hz
29	Risetti et al. (79)	14 DOC patients (7 VS, 7 MCS)	N100, MMN, and P300 10 electrodes (F3, Fz, F4, C3, Cz, C4, P3, Pz, P4, and Oz)	CRS-R	Auditory	N100 was delayed in VS compared to MCS patients. However, the presence of brain lesions might have accounted for the N100 latency delay observed in VS patients The MMN component of ERPs did not show significant differences in mean latencies and amplitudes between the two groups of patients P300 showed a significant delay in the VS compared to MCS patients. aHowever, P300 latency in VS with respect to MCS might be due to the different brain lesion between the two groups
**Concurrent EEG-TMS**						
30	Ragazzoni et al. (80)	13 DOC patients (8 VS/UWS, 5 MCS) 5 healthy participants	EEG-TMS 19 electrodes	CRS-R	Resting-State	The occurrence of TEPs in both ipsilateral and contralateral was observed in healthy control and MCS patients, but with a reduction of amplitudes for the MCS group. TEPs were restricted in the ipsilateral part in 3 of VS/UWS patients and absent in the other 5
31	Casali et al. (81)	12 DOC patients (6 VS/UWS, 6 MCS)	EEG-TMS perturbation complexity index	CRS-R	Resting-State	The perturbation complexity index during TMS was significantly lower for unconscious patients with respect to normal subjects
32	Gosseries et al. (82)	17 DOC patients (VS/UWS & MCS)	EEG-TMS high-density EEG	CRS-R	Resting-State	Distant waves with high frequency and low amplitude were observed in MCS patients after TMS, whereas adjacent waves with low frequency were observed in VS/UWS
33	Manganotti et al. (83)	6 DOC patients (3 VS/UWS, 3 MCS)	EEG-TMS 21 electrodes	CRS-R	Resting-State	Only one MCS patient showed long-lasting neurophysiological and behavioral modification during rTMS over the stimulated area
34	Bai et al. (84)	18 DOC patients (9 VS/UWS, 9 MCS)	EEG-TMS 62 electrodes	CRS-R	Resting-State	The excitability time in temporal and spatial domains increased between was significantly different between VS/UWS and MCS patients
35	Casarotto et al. (85)	81 DOC patients (43 VS/UWS 38 MCS) 150 healthy participants	EEG-TMS 60 electrodes	CRS-R	Resting-State & fractal dimension	Despite the ability to fully discriminate between consciousness and unconsciousness, no significant differences were found between VS and MCS cohorts

*The table shows the main results reported in the literature reviewed for the electrophysiological methods*.

## Diagnostic Tools

### Clinical Behavioral Examination

Although severities may vary from patient to patient, the common denominator in those suffering from disorders of consciousness is an absence of overt behavior. Accordingly, the very first methods used to diagnose DOC patients involved evaluations of behavior, with the aim of categorizing DOC severity based on the extent to which healthy behavior is lost. The Aspen Workgroup was the first to propose the diagnostic criteria for assessing the level of consciousness in patients with DOC, and their guidelines remain among the most important (86).

#### First Generation Scales

The first behavioral scale to be widely used for clinical DOC diagnosis was the Glasgow Coma Scale (GCS) (32). Developed in 1974 and initially used to assess the level of consciousness in patients after a head injury, the GCS was designed to test three behavioral categories, which include eye-opening, verbal response, and motor response. Today, it is still widely used in emergency rooms and intensive care units. However, this assessment scale is not sensitive enough to more subtle changes in consciousness that could differentiate VS/UWS and MCS patients. In fact, an evaluation of reliability and sensitivity for several behavioral scales showed that the GCS is prone to a high likelihood of misdiagnosis (87).

Another important early scale developed in 1979 was the Rancho Los Amigos Levels of Cognitive Functioning Scale (LCFS), which was designed specifically for evaluating cognition (88). The bottom three levels are used to describe patients who show limited responsiveness. Patients who are aware but confused would be classified in the next three levels, and the top two levels are assigned to patients who display appropriate behavior. Like the GCS, this first-generation scale is not reliable in detecting subtle differences that could be significant in differentiating DOC categories.

To address the reliability issues of the GCS and focus more on motor responses, the RLS85 was developed in 1988 (89). It is based on eight behavioral categories, known as reaction levels. The first level describes alertness, followed by levels 2 and 3 which describe drowsiness and confusion. Unconsciousness is first described in level 4, though in this stage the patient will still ward off pain. Levels 5–8 describe unconscious states based on a varying demonstration of motor stereotypes, with no response to stimulation at the highest level. Despite some improvements to earlier scales when released, the RLS85 guideline does not meet today's Aspen Workgroup diagnostic criteria.

#### Second Generation Scales

The second generation of behavioral scales was developed in the 1990s with the advent of the Coma Recovery Scale (CRS). Introduced by Giacino et al. in 1991 (90), the development of the CRS was motivated by the poor discriminatory performance of the GCS and LCFS. The main goal was to develop a more standardized methodology with tests that indicate the level of neurobehavioural function. To improve CRS further, a revised version (CRS-R) was proposed in 2004 (26). The revision was motivated by 12 years of clinical experience, previous analyses concerning the scale's psychometric characteristics, and changes in diagnostic parameters pertinent to DOC patients (26).

The CRS-R consists of 23 points obtained from six hierarchical subscales that include auditory, visual, motor, communication, and arousal functions. The sensory stimuli are administered in a standardized manner, and scoring is based on the presence/absence of specific behavioral responses. In each subscale, the lowest scores correspond to no behavioral response or reflexive activity, whereas higher scores are recorded if patients demonstrate cognitively mediated behaviors, such as functional object use or verbal communication. Although a large number of other tests exist, the CRS-R is the only one to meet all Aspen-Workgroup criteria for accessibility, standardization, and interpretative use (91). In a comparison study, 13 clinical scales were evaluated, and it was shown that the CRS-R is the most robust and reliable scale in identifying VS/UWS, MCS, and exit-MCS, a category used to describe patients emerging from MCS (91). As a result, the CRS-R has been the most recommended tool for assessing DOCs (26).

While the CRS-R has proven to be a valuable clinical tool, there are still limitations to its use. For instance, CRS-R scores can fluctuate with the time of day; scores on the visual and auditory subscales are higher in the morning compared to the afternoon in both VS/UWS and MCS patients (92). Furthermore, a study by Wannez et al. (93) found that behavioral fluctuations result in significant differences between the results of the first four CRS-R repetitions, and it was therefore recommended that this test be administered at least five times. This is a significant problem because accurate diagnosis is critical to making decisions regarding treatment selection, prognosis, and end-of-life decisions (94, 95). Studying DOC from a purely behavioral perspective is also problematic because with such assessments, it is not possible to study neural processes that are unrelated to or may be disconnected from behavior. Therefore, relying on other assessment tools, especially those that provide neurological biomarkers, may help address this issue and improve diagnostic accuracy.

Several other scales have also been used to diagnose DOC patients, though we do not discuss those in detail here. These include the Full Outline of Unresponsiveness (FOUR) (32), the Western Neuro Sensory Stimulation Profile (WNSSP) (28), the Sensory Modality Assessment and Rehabilitation Technique (SMART) (27), and the Wessex Head Injury Matrix (WHIM) (96).

### Neuroimaging Methods

Neuroimaging has provided unprecedented abilities to directly examine potential dysfunction in the brains of patients who have survived a traumatic brain injury. Examining structural and functional properties of the brain to infer disruptions in cognitive abilities allows for a more complete evaluation of a patient's mental state. From the available neuroimaging methods, PET and fMRI have proven to be particularly valuable in assessing cognitive processes (97) in DOC patients. In this section, we describe some important neurobiological developments obtained with PET and fMRI. For brief summaries of the studies we mention, see Table 2.

#### Positron Emission Tomography

PET is a functional imaging technique that is based on the intravenous injection of a radioactive tracer, such as water or a type of sugar. Metabolic brain measurements obtained with PET have allowed for the first evaluations of brain abnormalities in the cortical and subcortical areas. A seminal PET study (12) described a reduction of cerebral glucose metabolism by up to 60% in VS/UWS patients compared to normal rates. Further understanding of the neural mechanisms in the vegetative state was pursued in a 2000 paper that employed PET scans with a (15) O-radiolabeled water tracer to evaluate changes in cerebral blood flow (7). With auditory stimulation, they found reduced activity in the temporo-parietal junction cortex for VS/UWS patients compared to healthy controls. In addition, functional disconnections, as indicated by psychophysiological interaction analysis, were observed between the auditory cortex and other regions, particularly the posterior parietal association area, the anterior cingulate cortex, and the hippocampus. They concluded that the extent of these functional disconnections prevents VS/UWS patients from having normal integrative processes, which are critical to awareness (7).

Moving beyond investigations that compared DOC patients to healthy controls, more recent studies aimed to employ PET to identify differences between different types of DOCs, particularly VS/UWS and MCS patients. Stender et al. showed that FDG-PET could discriminate VS/UWS from MCS with an 85% agreement to CRS-R, which outperformed classification obtained with other methods at the time (fMRI yielded 63% correspondence to CRS-R) (13). They also found that FDG-PET was better at predicting outcomes for patients, with 74% outcome prediction accuracy compared to 56% for fMRI. However, this study was based on an approach that only compared the relative metabolic rates of different brain regions between groups. To obtain more complete indicators and establish more robust diagnostic guidelines, it was necessary to incorporate absolute global comparisons for the entire brain, as these can provide important information on functional pathways. Therefore, the same researchers aimed to quantify absolute differences in brain glucose metabolism to distinguish DOC severity. By measuring the global cerebral metabolic rate of glucose (CMRglc) and using machine learning classifiers, they found that absolute cortical metabolism can differentiate VS/UWS from MCS with 82% accuracy (36). These results provide strong evidence that FDG-PET is a viable diagnostic tool for DOC patients.

PET studies have also employed noxious stimulation, which is commonly achieved by administering electric shocks in the median nerve (hand). In an experiment involving healthy and DOC subjects, neural activation of the pain processing network was shown to be equivalent between MCS patients and healthy controls. For all regions examined, MCS patients had higher activation than patients in a persistent vegetative state (98). Beyond emphasizing an important distinguishing feature between the two DOC groups, the knowledge that MCS patients can perceive pain also had significant implications on our understanding and treatment of the condition.

Finally, recent work has underscored the advantage of employing PET alongside EEG. In a study with 21 VS/UWS and 31 MCS patients, Hermann et al. (38) compared an FDG-PET measure known as the metabolic index of the best-preserved hemisphere (MIBH) with quantitative EEG (qEEG) metrics that were previously known to correspond with CRS-R diagnosis (see section Quantitative EEG for more details on qEEG). While neither method alone was significantly more accurate, it was found that combining the data of FDG-PET and EEG in a classification procedure allowed for significantly improved classification; in classifying patients from the two groups, the positive and negative predictive values for identifying MCS were 81 and 88%, respectively (38).

Undoubtedly, critical knowledge has been gained on disorders of consciousness by analyzing metabolic activity with PET. Despite these results, there are unavoidable limitations to this technique. One such drawback relates to the use of a radioactive tracer. Although exposure to harmful radiation is minimal, it still poses a safety concern to some. In addition, severe head injuries can obstruct the blood-brain barrier, which can lead to a change in the normal relationship between neuronal metabolic and hemodynamic activity that PET relies on, thus resulting in a source of error in acquisition (99).

#### Functional Magnetic Resonance Imaging

Several fundamental discoveries about the magnetic properties of blood led the advent of fMRI, which has become an invaluable tool for examining neural mechanisms of the brain (100). The critical juncture in these developments was the detection of the blood-oxygen-level dependent (BOLD) signal (101). Through BOLD, each voxel (a three-dimensional pixel of the image) obtains a time-series which allows fMRI to map cerebral activity over time.

In the mid 2000s, fMRI became more widely used in studies involving DOC patients (102). Such studies have employed two broad categories of fMRI evaluations. One category is event-related fMRI (er-fMRI), where brain activity is recorded while individuals complete a task. The second category is resting-state fMRI (rs-fMRI), which requires no task, and the brain is hence described as being at “rest.” This type of analysis allows for the assessment of intrinsic brain functionality. The following two subsections describe studies employing er-fMRI and rs-fMRI in DOC populations.

##### Event-Related—fMRI

As with PET, fMRI studies first aimed to investigate the neural mechanisms underlying residual cognitive abilities in patients with severe brain injuries. Once it was established that fMRI can be a useful tool in identifying VS/UWS cognitive processes, this technique was used in an attempt to differentiate VS/UWS and MCS patients (103). By measuring activity in response to subjects hearing their name, it was found that all four of the MCS patients and two of the seven VS/UWS patients (who were notably the only ones who later improved to MCS) showed higher-order processes in the areas of the temporal-lobe. The results obtained with fMRI confirmed those of previous PET studies, which gave merit to this technique in evaluating DOC (103). Although this study was limited by a small patient sample and some inconsistencies within the VS/UWS group, it motivated subsequent work that employed fMRI to investigate how activity patterns of different brain regions vary between VS/UWS and MCS individuals.

One such study was conducted in 2011 by Crone et al. who employed a passive sentence-listening task with VS/UWS and MCS patients as well as healthy controls (40). Patients listened to short sentences with true and false statements, and the goal was to observe whether they could modulate speech processing based on the sentence's content. It was found that when arousal is normally expected, the medial prefrontal region was not activated for VS/UWS patients. Activity in the same region was present in MCS patients but still reduced compared to healthy controls (40). Therefore, the overall results indicated a gradient in brain activity that corresponded to DOC severity. In a subsequent study, brain activity in response to music was compared between MCS and VS/UWS patients (41). Here, the regions of interest were the bilateral superior temporal gyri (STG), which were activated in both healthy controls and MCS patients, while no activations were induced by the same stimuli in four of the five VS/UWS subjects (41). Notably, the single VS/UWS patient who showed similar STG activation to MCS and healthy subjects transitioned to an MCS diagnosis 4 months after the investigation (41).

These works showed that fMRI activity patterns observed during auditory stimulation may help predict recovery from VS/UWS to MCS. This was further explored in a 2017 study by Marino et al. where an fMRI listening task was used to classify patients as “VS Converted,” “VS Stable,” and MCS, in which the former two groups represent VS patients who converted to MCS and those who did not, respectively (42). The common feature that separated “VS Stable” from the other categories and helped predict a transition to MCS was a high activation of the primary auditory cortex. The authors emphasized that these features cannot be detected with behavioral scales, hence stressing the prognostic value of fMRI in the clinical evaluation of DOC.

Moving beyond passive tasks where a stimulus is presented, others investigated whether patients diagnosed with DOC could follow commands. In one important study (4), fMRI was used on a single VS/UWS patient who was given mental imagery commands, which included playing tennis or navigating a familiar environment. They found that this patient was able to imagine playing tennis by producing activity in the supplementary motor area, as well as navigating their home by producing activity in the parahippocampal gyrus, posterior parietal-lobe, and lateral premotor cortex. The results indicated that despite the VS/UWS diagnosis, the individual was able to understand and respond to commands in a very similar way to healthy participants. This was the first result to demonstrate that DOC patients can accurately respond to commands with no overt behavior, highlighting that mental imagery paradigms are ideal for assessing residual cognitive and conscious processes in this patient population.

These ideas were later used to establish a comprehensive paradigm based on spatial navigation and motor imagery, which allowed for an accurate fMRI assessment of a patient's ability to voluntarily participate in such tasks (104). In 2013, Vogel et al. employed this paradigm in a study involving 10 VS/UWS and 12 MCS patients. The results for VS/UWS patients were promising, as the five of ten who showed significant BOLD signals in the region of interest emerged to MCS, whereas the other five showed no substantial activations and remained in the vegetative state. In other words, the paradigm had 100% specificity and sensitivity for the sample of VS/UWS patients (39).

##### Resting-State fMRI

In addition to using er-fMRI, resting-state fMRI (rs-fMRI) has been extensively used to assess neural mechanisms in patients diagnosed with DOC. As the nomenclature implies, rs-fMRI has the advantage of not requiring patients to engage in a task.

Many works involving rs-fMRI involve resting-state networks (RSNs), which are regions of correlated activity that reflect the brain's functional architecture. RSNs are characterized by low frequency (0.1 Hz) spontaneous fluctuations of the BOLD signal. The most widely discussed RSN is the Default Mode Network (DMN), which includes the medial prefrontal cortex, posterior cingulate/precuneus, superior temporal cortex, hippocampus, and inferior parietal cortex. The DMN was defined and popularized by Raichle et al. (105), and the synchronization of the DMN's constituent regions was confirmed with fMRI in 2003 (106). The DMN has often been described in two ways: (1) Positive DMN connectivity, corresponding to a positive correlation between intra-network components, and (2) Negative DMN connectivity, corresponding to negative correlations between the DMN and other networks.

The DMN has been extensively investigated in DOC patients. In a 2009 study, this was the network of interest for a patient who had been diagnosed as VS/UWS 2.5 years prior (107). It was found that brain regions associated with the DMN displayed a similar but reduced level of functional correlation compared to healthy individuals, supporting the hypothesis that although consciousness plays a role in the DMN, there are also unconscious mechanisms that can give rise to this functional network in VS/UWS patients (107).

In the following year, Vanhaudenhuyse et al. investigated the DMN using rs-fMRI on VS/UWS and MCS patients. An exponential correlation was reported between positive DMN connectivity and the loss of consciousness, with VS/UWS patients showing the most hypoconnectivity (44). These results were extended in a 2013 study, which further confirmed that symptom severity (VS/UWS vs. MCS) corresponds to hypoconnectivity of the DMN's regions, and that these regions are hyperconnected to external limbic structures (45). Overall, these studies emphasize that even if the DMN can be detected in the VS/UWS condition, the extent to which it is active and connected to other structures can be a factor in helping clinicians differentiate VS/UWS and MCS patients.

Beyond the DMN, several other RSNs that could represent DOC biomarkers have been identified. These include the Salience Network (SN: frontal cortex, anterior cingulate, and anterior insular cortex circuitry), the Dorsal Attention Network (DAN: insular cortex and posterior parietal), the Auditory Network (AN: temporal cortex), the Sensorimotor Network (SMN: striatal and parietal cortex), and the Visual Network (VN: occipital cortex) (108). In a notable 2015 study involving DOC patients in Liège, Belgium, Demertzi et al. applied machine learning to compare different RSNs in their ability to distinguish VS/UWS, MCS, and coma patients. It was found that differences in all the aforementioned networks could differentiate the two conditions with at least 80% accuracy. Of these, the auditory network gave the highest accuracy, with 20 out of 22 patients classified in congruence with behavioral scales (109). These encouraging results emphasize that the conjuncture of rs-fMRI with machine learning can be a very accurate diagnostic tool for DOC.

This work was succeeded by other machine learning-based research on the role RSNs have in mediating consciousness, with more focus given to how these networks might interact. By examining rs-fMRI data and applying a topological correlation analysis on VS/UWS and MCS patients, Long et al. discovered abnormal connectivity patterns between the DMN and the FPN that could separate the two DOC groups with 82.7% accuracy (47). Other RSNs have shown further differences between VS/UWS and MCS patients, such as the left executive control network, which was found to be impaired in VS/UWS but preserved in MCS (43).

Studies with rs-fMRI also extended to broader, whole-brain activity analyses that are not necessarily limited to individual RSNs. In 2019, Demertzi et al. (51) studied several features related to BOLD signal coordination over the entire cortex with healthy controls, VS, and MCS patients. More specifically, activity coordination was analyzed with metrics such as phase synchronization and coherence to help detect patterns associated with different conscious states. One pattern, related to long-range inter-region coherence, was found to be most prevalent in healthy controls and more likely to occur in MCS than VS/UWS patients. Notably, a pattern characterized by low coordination/coherence was found to be highest in the VS/UWS group, providing a possible distinguishing feature between the two DOC categories (51).

Recently, it has also been proposed that resting-state neuronal dynamics can be characterized with chaotic features, such as fractal dimension and entropy. This was attempted in both healthy (110) and pathological conditions (52, 111). Entropy and fractal dimension can be seen as statistical measures of signal complexity, and these are further discussed below in the section on EEG. In 2020, Varley et al. (52) showed that when extracted from functional connectivity data, fractal dimension could differentiate healthy controls and DOC patients as well as MCS and VS/UWS subjects, with higher fractal dimension being associated with the milder symptoms seen in MCS (52).

There is little doubt that fMRI has helped improve diagnostic accuracy by identifying functional brain network dynamics associated with disorders of consciousness. Unfortunately, there are disadvantages to using fMRI. First, patients may feel uncomfortable or even claustrophobic due to the small space of the MRI bore. There are also restrictions for patients with specific health conditions, especially those that use medical implants such as pacemakers. Moreover, motion artifacts induced by head movements during scanning are common for DOC patients, which can introduce significant flaws in image acquisition. This has led experts to suggest multiple scanning sessions for patients (112), even though doing so could increase the chance of patients feeling discomfort in the scanner. From a practical perspective, patients must be transferred to a suitably equipped PET/fMRI facility, and this can be a challenging demand for studies that require frequent reassessments. Finally, PET and fMRI may not be suitable for patients in the acute stage due to factors related to critical care, such as ventilation. Unquestionably, the main disadvantages related to PET and fMRI as analysis tools for DOC have to do with the incompatibility of these techniques at the bedside.

### Electrophysiological Methods

The necessity of measuring detectable neurobiological information at the bedside has encouraged the employment of electrophysiological techniques such as an electroencephalogram (EEG) to DOC patients (3). The relative portability of these methods allows for their rapid employment during critical phases in the intensive care unit, and they can be employed repeatedly with relative ease (55). In this section, we overview literature that investigates how EEG can be used to assess DOC. The main electrophysiological techniques these studies employ are summarized in Figure 2, and the papers we cite are summarized in Table 3.

#### Electroencephalography

EEG measures the brain's electrical activity directly and non-invasively. Specifically, the activity recorded by EEG electrodes reflects the sum of post-synaptic potentials of synchronized cortical pyramidal neurons at their apical dendrites (113). The high sampling rate of EEG allows for signal acquisition with high temporal precision, and as a result, these measurements can yield very important information about patterns of brain activity in different neurological conditions (114, 115).

This section explains how different forms of EEG measurement allowed for increased understanding and improved diagnosis of DOC (93). The first section centers on Event-Related Potentials (ERPs), which involve sensory or cognitive processes in response to some event, such as the presentation of an image or a sound (116). The second and third sections are based on quantitative EEG (qEEG), a computational approach aimed at quantifying several features related to a signal's neural dynamics. The methods covered here include power spectrum analysis, whole-brain functional connectomes [such as during resting-state EEG (rs-EEG) (117–119)], the bispectral index (BIS) (120, 121), Entropy Analysis (E), and Fractal Dimension (FD) (122).

##### Event-Related Potentials

An Event-Related Potential (ERP) corresponds to neural activity recorded at specific electrode locations over the scalp in response to sensory, cognitive, or motor events. From these events, electrical activity can be used to generate ERP waveforms that are commonly described using latency (time) and amplitude. The components are usually named with a letter representing positive or negative amplitude polarity (P/N), followed by a number representing the expected latency time in milliseconds (ms). For example, the N100 is characterized by a negative potential and an average latency of 100 ms (although it can be between 70 and 140 ms). As one would expect, different ERPs correspond to different stimuli and tasks.

Early studies used ERPs to investigate residual cortical activity in VS/UWS patients. This was done in 2004 study by Schöenle and Witzke, who focused on the N400, an ERP component previously observed in healthy patients upon hearing semantically incoherent sentences in a sentence-listening task (123). They found that almost 40% of the VS/UWS group displayed some level of the N400 component associated with anomalous sentences, with 12% of the patients showing waveforms similar to those seen in healthy controls (123). Their results suggested preserved semantic processing in some patients diagnosed as VS/UWS, which was previously impossible to detect with clinical behavioral scales. This highlighted the value of ERPs, and particularly the N400, as tools that can assess the cognitive capabilities of DOC populations.

In 2013, Balconi et al. (74) extended this to examine semantic processing in both VS/UWS and MCS patients. By presenting incongruous sentences much like the previous study, they confirmed that both patient groups produced an N400 with a greater latency relative to healthy controls. However, no distinguishing factors could be found between the VS/UWS and MCS groups with this ERP (74). Despite no differences between the two severities, the N400 did provide clinically relevant information, such as the ability to evaluate semantic associations in VS/UWS individuals. In 2013, a positive correlation was found between the N400 response and the recovery of patients, suggesting the potential use of this signal as a prognostic indicator (124). However, this result has been subject to debate (125, 126). In particular, Cruse et al. who manipulated the task used to activate the N400, showed that changes to stimuli and task demands significantly influence the probability of detecting statistically significant N400 effects (126). However, the authors concluded that this does not necessarily diminish the merit of N400 in DOC studies. Rather, attention must be paid to the types of tasks that elicit significant sensitivity to this measure.

Besides the N400, several other ERPs have been investigated in studies aiming to differentiate VS/UWS and MCS patients. A 2005 paper by Kotchoubey et al. (71) investigated residual ERPs in both VS/UWS and MCS patients, with a focus on N100, P100, P200, P300, and mismatch negativity (MMN, see below). Interestingly, the N100 component was found to be preserved in VS/UWS patients but delayed in the MCS group, suggesting that the two groups differ in their responses with short latency ERP components (79). However, there was significant heterogeneity within patient groups when observing these ERP components, indicating that the N100 and P100 components are unlikely to differentiate VS/UWS from MCS patients (71, 79).

Further research on DOC was based on stimuli and tasks that evoke responses with longer latency, which are considered to reflect higher-order cognitive processes (125). One such component is mismatch negativity (MMN), which represents the brain's automatic processing of a stimulus difference. It is elicited by the so-called “oddball paradigm,” a method for auditory stimulation consisting of a common sound and a similar but different sound that is less frequent (such as a deviant “boop” in a series of “beeps”) (127). It was proposed that changes in MMN amplitude and latency were associated with symptom severity and outcomes in VS/UWS patients (63, 128). The P300, also associated with the oddball paradigm, is another ERP component that fits the category of longer latency (129). ERPs in oddball tasks have been shown to vary depending on the relative complexity of stimuli, which was observed through the modulation of P300 latency for more complex semantic processing (higher-level processing was associated with longer latency) (129). This motivated the comparison of P300 modulation between VS/UWS and MCS patients in a 2011 study by Cavinato et al. Using varied stimulations, no modulation of P300 was observed in VS/UWS patients. However, MCS patients and healthy controls demonstrated P300 latency modulation with varied stimulus complexity, providing an important distinction between the two patient groups (62).

As seen with the ERP components mentioned above, there are still ambiguities surrounding their efficacy in distinguishing DOC severity. Undoubtedly, ERP analysis has been crucial in studies of DOC populations as they allowed for the discovery of residual cognitive functions that were previously believed to be absent. On the other hand, attempts to identify specific conditions of unresponsiveness have yielded inconsistent results. The most promising analyses were those involving the N400, P300, and MMN, which should be the focus of further research.

##### Quantitative EEG

The implementation of more complex numerical computations in waveform analysis gave rise to qEEG. Power spectrum analysis, which is used to study the frequency content of an EEG signal, has been extensively used to study DOC populations (58, 122). This analysis involves specific categories of neural oscillations, which are rhythmic waveforms at specific frequencies. The most common waveforms are the delta, theta (low frequency), alpha (low-intermediate frequency), beta, and gamma (high frequency). In general, qEEG allows for the analysis of more complex signal properties and interactions, making it a potentially powerful tool for research involving DOC patients.

In 2012, Fingelkurts et al. conducted a series of studies using short-term EEG spectral patterns with patients in a resting-state condition (rs-EEG). They aimed to identify differences in spontaneous activity between VS/UWS and MCS groups (54). With probability-classification analysis, they found that the likelihood of occurrence for the delta, theta, and slow alpha oscillations was higher in the VS/UWS group relative to MCS (54, 130). The results confirmed previous observations that a loss of consciousness is associated with a more frequent occurrence of slow EEG oscillations. Conversely, they also found that fast alpha oscillations had a higher probability of occurring in MCS patients (54).

Other works focused on comparing the overall power of these oscillations and how different regions of the brain are connected through them. It has been confirmed that VS/UWS patients show increased delta power and decreased alpha power compared to MCS patients (21, 59, 60). Extended analysis revealed that connectivity through the theta and alpha bands is significantly higher in MCS patients, demonstrating a correlation between this measure and patients' CRS-R scores (55, 131, 132). These results were encouraging, as theta and alpha synchronization between regions is associated with higher-order cognitive processes (131). More recently, features extracted with EEG have been used in extensive machine learning procedures to differentiate MCS and VS/UWS patients, and these methods were shown to be robust across a wide range of clinical settings and acquisition methodologies (58).

These studies have also highlighted the usefulness of analyzing whole-brain rs-EEG connectivity, which can give valuable insight into large-scale brain dynamics that may be associated with consciousness. A recent publication described how this technique could be used to characterize the whole-brain topological properties of DOC patients. By employing rs-EEG on VS/UWS and MCS subjects, the authors identified quantitative network properties with significant differences between the two groups, with the conclusion that these differences could have diagnostic value (133). From a network perspective, consciousness has been associated with a high degree of integration between the brain's subnetworks (134). Accordingly, this work characterized the VS/UWS brain as consisting of more minor, disconnected networks that do not contribute to higher integrative processes (133).

Another important qEEG metric is the bispectral index (BIS), which was initially developed for anesthesia monitoring (120, 121). This parameter is obtained using a weighted sum of several EEG features in both the time and frequency domains, with values ranging from 0 (isoelectric EEG) to 100 (normal activity). In a multicentre study that evaluated the ability of several EEG parameters to distinguish VS/UWS and MCS patients (57), the BIS yielded the most significant difference between the groups. This metric correlated strongly with CRS-R scores, as significantly lower BIS values corresponded to the VS/UWS condition (57). Furthermore, the same study found higher BIS values in patients who recovered at 1-year post-injury as compared to patients who did not. The authors explained these results by arguing that since BIS is a complex measurement that involves the integration of several EEG parameters, it is more effective as a classifier than more simple features that could not achieve differentiation (57). Finally, one more metric that may also be part of qEEG analysis is Fractal Dimension (122). However, it has not yet been used in attempts to differentiate VS/UWS and MCS patients, so this could be another potential measure for future studies to experiment with.

##### Non-linear EEG Approaches

In addition to linear methods, non-linear approaches can be useful tools for examining irregular and non-periodic electrophysiological patterns (110, 111, 119) in both healthy and pathological brains (135). Entropy and fractal analysis are non-linear processing techniques derived from chaos theory which are increasingly used to analyse EEG signals (119, 135–137). Additional evidence has demonstrated that in many cases, EEG waveforms do not reflect periodic behavior, but rather a brief period of activity that is repeated intermittently (non-rhythmically) (138, 139). Recognizing the nature of these time series and using nonlinear processing techniques may uncover hitherto overlooked physiological information in DOC populations (111, 140–142).

Different approaches can be applied to measure the entropy of EEG signals, which effectively quantifies the signal's degree of complexity and irregularity (143). These include approximate entropy, Lempel–Ziv complexity, permutation entropy, and Kolmogorov–Chaitin complexity (67, 144). Generally, higher entropy is associated with more stochastic EEG signals, reflecting more active information processing. This is seen when one compares the entropies recorded during wakefulness and sleep. Accordingly, healthy individuals were found to have higher values of entropy compared to DOC populations, while MCS individuals had higher entropy than VS/UWS patients (59, 145). These entropy measures correlated with CRS-R scores and in some cases were shown to be more effective than BIS in discriminating the two conditions (59).

#### Concurrent TMS-EEG

Another advantage gained from the compact nature of EEG is that it can be combined with other tools, such as Transcranial Magnetic Stimulation (TMS). This led to the development of important techniques that can be useful when working with DOC patients.

The combination of EEG and TMS (TMS-EEG) in a cognitive experiment allows for the observation of ERPs that follow cortical stimulation from TMS, known as TMS Evoked Potentials (TEPs) (146). In a 2014 study, TMS-EEG was shown to be a reliable tool in discriminating states of consciousness (147). Another study compared VS/UWS and MCS patients using TEPs, traditional ERPs, and somatosensory-evoked potentials (SEPs), of which TEPs yielded the most significant differences between the groups (80). This was seen in both the amplitude (reactivity) and distribution (connectivity) evoked by TMS stimulation of the primary motor cortex. TEP measurements were closer to normal in MCS patients but severely impaired in VS/UWS patients, suggesting that cortical reactivity and connectivity were significantly damaged in the latter group (80).

Another study with promising results was conducted by Casarotto et al. (85), who attempted to measure consciousness by treating TMS-EEG data with a mathematical analysis. They hypothesized that consciousness depends on the brain's ability to support complex activity patterns through the integration and differentiation of information in time and space domains. Accordingly, they introduced a metric called the perturbational complexity index (PCI), which involves a complex analysis of electrocortical responses to perturbation with TMS. Impressively, PCI was able to categorize MCS patients with a 94.7% accuracy. It also allowed for the identification of a small but substantial portion of the VS/UWS patients who had high PCI values, indicating the possibility for some level of consciousness that cannot be observed behaviourally (81). Despite these promising results, TMS-EEG is not yet available for clinical purposes. However, further research and developments should be pursued for its eventual implementation in clinical settings.

## Summary and Conclusion

In this paper, we provided a comprehensive overview of the research devoted to better understand and diagnose the vegetative and minimally conscious states. These efforts started with the development of clinical behavioral scales, of which the most popular has been the CRS-R.

To address the accuracy issues of behavioral scales in differentiating VS/UWS and MCS patients, substantial progress has been made to incorporate advanced neuroimaging and electrophysiological tools. With PET, meaningful metabolic differences in the frontoparietal cortex and thalamus were found between VS/UWS and MCS patients during motor and visuospatial imagery tasks (13).

Nowadays, fMRI has outgrown PET due to the lack of radioactive materials and the advantage of high spatial resolution brain mapping. Several fMRI studies we mentioned have focused on the detection of resting-state networks and their association with different levels of consciousness (4, 41, 42). Most notably, the DMN showed high sensitivity in discriminating between VS/UWS and MCS patients (45, 49). It was also shown that connectivity between DMN and other brain networks (i.e., frontoparietal cortex) is likely altered in patients diagnosed with DOC, as the functional connectivity unexpectedly shifts toward limbic structures (48). Despite these important findings, fMRI is limited by its lack of portability, high costs, and inclusion criteria, all of which prevent it from being an ideal clinical tool.

The difficulties associated with neuroimaging techniques such as PET and fMRI make EEG another suitable diagnostic candidate. EEG is a highly portable system that does not impose undesirable effects on patients. Our discussion on EEG started with the Event-Related Potentials (ERPs), among which the MMN and P300 seem to be the most promising diagnostic metrics. In studies involving the oddball paradigm, modulation of P300 latency in response to stimulus complexity seems to be the most promising feature that could differentiate between VS/UWS and MCS patients (62, 63, 79, 148).

Important results were also obtained from resting-state EEG (rs-EEG) studies, several of which have shown high accuracy in discriminating between VS/UWS and MCS patients (55, 60, 130, 131, 149). Among these, the most commonly used metrics have been the power spectrum, the bispectral index, and entropy, which also showed an ability to predict a patient's recovery (60). Furthermore, the combination of TMS and EEG has emerged as a very promising methodology for examining the brains of patients with DOC. Simultaneous administration of EEG and TMS can yield important information without explicitly asking the patient to perform a specific task, which helps avoid issues related to commands in standard behavioral tests (80, 82, 85).

Whether employing fMRI, PET, or EEG, there are multiple factors that can affect a patient's responsiveness to a task and their corresponding brain activity patterns. These include medications, fatigue, sleeplessness, as well as the patient's interest and motivation. Moreover, many of the paradigms discussed require participants to have intact language abilities to understand instructions. For these reasons, there are valid concerns about the reliability of task-based DOC studies.

Although there are promising foundations to develop standardized diagnostic guidelines for DOC with neuroimaging and EEG, the ultimate solution to understanding and diagnosing these disorders transcends the technologies we described. One of the biggest challenges in many of these studies is collecting a substantial patient sample size to perform meaningful comparisons. Therefore, it would be greatly beneficial for researchers to have access to shared databases with organized fMRI, PET, EEG, and behavioral scale data from DOC patients. The importance of this is emphasized by the multifaceted and interdisciplinary nature of this problem, especially for contemporary efforts that seek to improve diagnostic accuracy by combining different imaging methods [i.e., FDG-PET and EEG (38)]. Ultimately, we believe that the scientific and medical communities will only be able to reach a valid consensus on DOC guidelines if results and data from different studies share a similar foundation. Having this sort of database will also make it easier for more experimentation and trials by different research groups, all of which will help us better understand the disorders of consciousness that dreadfully impact so many people's lives.

## Author Contributions

All authors listed have made a substantial, direct, and intellectual contribution to the work and approved it for publication.

## Conflict of Interest

The authors declare that the research was conducted in the absence of any commercial or financial relationships that could be construed as a potential conflict of interest.

## Publisher's Note

All claims expressed in this article are solely those of the authors and do not necessarily represent those of their affiliated organizations, or those of the publisher, the editors and the reviewers. Any product that may be evaluated in this article, or claim that may be made by its manufacturer, is not guaranteed or endorsed by the publisher.
